# Bidirectional association between serum carcinoembryonic antigen and metabolic syndrome among the Chinese male population: two cohort studies

**DOI:** 10.1186/s12944-020-01411-7

**Published:** 2020-11-04

**Authors:** Yafei Liu, Zhaohui Du, Jiadong Ji, Jingru Li, Deming Bi, Fang Tang

**Affiliations:** 1 Center for Big Data Research in Health and Medicine, The First Affiliated Hospital of Shandong First Medical University & Shandong Provincial Qianfoshan Hospital, Jinan, China; 2 Shandong Provincial Qianfoshan Hospital, Cheeloo College of Medicine, Shandong University, Jinan, China; 3 Fred Hutchinson Cancer Research Center, Seattle, WA 98109 USA; 4 Department of Data Science, School of Statistics, Shandong University of Finance and Economics, Jinan, China; 5 School of Public Health, Weifang Medical University, Weifang, China; 6 Department of Surgery, Zhangqiu District Hospital of Traditional Chinese Medicine, Jinan, China

**Keywords:** Carcinoembryonic antigen, Metabolic syndrome, Cohort study, Bidirectional association, Smoking, Hazard ratio, Cox proportional hazards model

## Abstract

**Purpose:**

Previous studies have shown that serum carcinoembryonic antigen (CEA) is independently associated with metabolic syndrome (MetS). However, these studies were mainly cross-sectional analyses, and cause was not clarified. In the present study, two bidirectional cohort studies were conducted to investigate the bidirectional associations between CEA and MetS using a Chinese male sample cohort.

**Methods:**

The initial longitudinal cohort included 9629 Chinese males enrolled from January 2010 to December 2015. Two bidirectional cohorts were conducted in the study: subcohort A (from CEA to MetS, *n* = 6439) included participants without MetS at baseline to estimate the risk of developing incident MetS; subcohort B (from MetS to CEA, *n* = 8533) included participants without an elevated CEA level (Hyper-CEA) at baseline to examine the risk of developing incident Hyper-CEA. Hazard ratios (HRs) and 95% confidence intervals (CIs) were estimated using Cox proportional hazards models.

**Results:**

In subcohort A, the incidence densities of MetS among participants with and without Hyper-CEA were 84.56 and 99.28 per 1000 person-years, respectively. No significant effects of Hyper-CEA on incident MetS were observed in subcohort A (HR, 0.89; 95% CI, 0.71 to 1.12; *P* = 0.326). In subcohort B, a higher incidence density of Hyper-CEA was found among participants with MetS (33.42 and 29.13 per 1000 person-years for those with and without MetS, respectively). For nonsmoking participants aged > 65 years, MetS increased the risk of incident Hyper-CEA (HR, 1.87; 95% CI, 1.09 to 3.20; *P* = 0.022).

**Conclusion:**

For the direction of CEA on incident MetS**,** no significant association was observed. For the direction of MetS on incident Hyper-CEA, MetS in nonsmoking elderly men could increase the risk of incident Hyper-CEA, while this association was not found in other stratified participants. The clinical implications of the association between CEA and MetS should be interpreted with caution.

**Supplementary Information:**

The online version contains supplementary material available at 10.1186/s12944-020-01411-7.

## Introduction

Metabolic syndrome (MetS) is a combination of metabolic abnormalities including hypertension, obesity, hyperglycaemia and dyslipidaemia and is associated with a greater risk of developing type 2 diabetes, cardiovascular disease, hepatic steatosis, and other circulatory disorders [1]. Many studies have found that MetS can also increase cancer risk, especially for colorectal, breast and prostate cancer [2, 3]. Serum carcinoembryonic antigen (CEA) is widely recognized as a serological tumour marker [4]. It is also expressed in nonmalignant conditions, such as ageing, smoking, chronic renal failure and some chronic inflammatory disease [5]. Previous studies have found serum CEA level can affect cardiometabolic diseases, including acute coronary syndrome [6], carotid atherosclerosis [7], diabetes [8–10], and obesity [11, 12].

Insulin resistance, a primary mechanism of MetS [13], is also associated with CEA level [12]. Hyperinsulinemia (a hallmark of insulin resistance), increase in bioavailable insulin-like growth factor I and the overproduction of reactive oxygen species appear to have a role in tumour initiation [14]. One study found that among Korean nonsmoking females, CEA was associated with MetS [15]. Another study showed that MetS and its components significantly increase according to the quartile of serum CEA concentration [16]. In this study, there was a positive association between CEA and MetS risk (Supplemental Table S1 and S2) based on a cross-sectional study, consistent with a previous study. The previous studies were cross-sectional, and the cause and effect association between MetS and CEA was limited.

In the present study, two bidirectional longitudinal cohorts were conducted to determine the cause and effect association between MetS and CEA: subcohort A (from CEA to MetS) and subcohort B (from MetS to CEA), both of which were based on large-scale health check-ups among northern urban Han Chinese males.

## Materials and methods

### Study population and data collection

Data were obtained from electronic medical records of a routine health check-up programme in the Center for Health Management of Shandong Provincial Qianfoshan Hospital. The study was approved by the Ethics Committee of Shandong Provincial Qianfoshan Hospital (No. for IRB approval: [2018] S0056). Written informed consent was obtained from all participants.

### Participants selection

#### Initial cohort

**Fig. 1:**
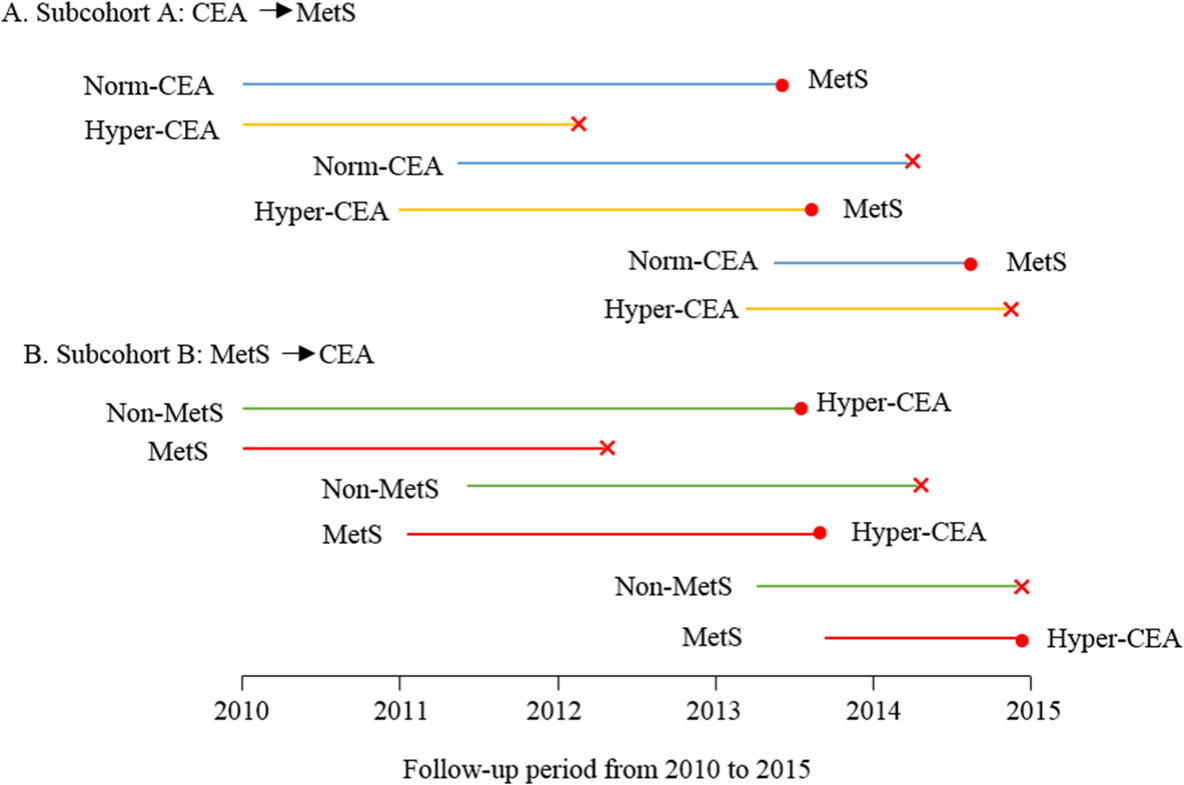
Diagram of the bidirectional longitudinal cohort. A: Subcohort A (from CEA to MetS, *n* = 6439) includes men with or without Hyper-CEA (≥ the upper limit of normal) at baseline to follow-up the incidence of MetS; B: Subcohort B (from MetS to CEA, *n* = 8533) includes men with or without MetS at baseline to follow-up the incidence of Hyper-CEA

Participants were eligible for initial inclusion if they were male, > 20 years old and had at least two health check-up records in more than 1 year from January 2010 to December 2015. Participants who had underlying medical conditions at baseline were excluded, including chronic liver disease, chronic renal disease, thyroid dysfunction, chronic inflammatory disease (e.g., chronic obstructive pulmonary disease and gastroenteritis), occupied lesions or cancer. Individuals with abnormal renal function (serum creatinine ≥115 μmol/l) or abnormal hepatic function (serum aspartate aminotransferase [AST] > 100 U/l or alanine aminotransferase [ALT] > 100 IU/l) were also excluded in this study. Based on the initial cohort, two subcohorts (subcohort A and B) were conducted to investigate the bidirectional association between CEA and MetS (Fig. 1). 9629 participants were included in the initial cohort.

##### Subcohort A

Subcohort A was conducted based on the initial cohort. Participants who had cardiovascular disease or MetS at baseline were further excluded from the initial cohort. Overall, 6439 participants were included in subcohort A.

##### Subcohort B

Subcohort B was conducted based on the initial cohort. Participants with elevated serum CEA (Hyper-CEA) at baseline were excluded from the initial cohort. Overall, 8533 participants were included in subcohort B.

#### Measurements

All participants in the initial cohort underwent a general health questionnaire and anthropometric and laboratory testing in Shandong Provincial Qianfoshan Hospital. Body weight and height were measured by standardized procedures when the participants wore light clothes and without shoes. Body mass index (BMI) was calculated as weight (kg) divided by height squared (m^2^). Blood pressure was measured on the right arm by an automated sphygmomanometer after a 5-min rest.

The related biomarkers, including fasting plasma glucose (FPG), total cholesterol (TC), triglyceride (TG), low-density lipoprotein-cholesterol (LDL-C), high-density lipoprotein cholesterol (HDL-C), alanine aminotransferase (ALT), aspartate aminotransferase (AST), gamma-glutamyl transpeptidase (GGT), blood urea nitrogen (BUN), serum creatinine (CREA), blood uric acid (BUA), haemoglobin (HB), white blood cell count (WBC) and CEA, were measured by the laboratory specialists using standard clinical and laboratory protocols. CEA was measured by two automatic immunoassay analysers in the hospital: Abbott i2000 (Chicago, USA; the reference of CEA is (0–5) ng/ml) and Johnson Vitros 3600 (Rochester, New York, USA; the reference is (0–3) ng/ml). All the biomarkers were measured in the Center for Health Management of the hospital.

##### Definitions of MetS

MetS was defined as the presence of three or more of the following five risk factors [17]: (1) abdominal obesity (waist circumference ≥ 85 cm). Because waist circumference was not available in this dataset, BMI was used to define obesity (BMI ≥ 25.0 kg/m^2^), which was recommended by the Diabetes Society of the Chinese Medical Association [18]; (2) elevated blood pressure (systolic blood pressure (SBP) ≥ 130 mmHg or diastolic blood pressure (DBP) ≥ 85 mmHg or with a history of hypertension); (3) elevated FPG (FPG ≥ 5.6 mmol/l (100 mg/dl)) or with a history of diabetes; (4) elevated TG (TG ≥ 1.7 mmol/l (150 mg/dl)); (5) reduced HDL-C (HDL-C < 1.0 mmol/l (40 mg/dl)).

##### Definitions of hyper-CEA

“Hyper-CEA” was defined as a positive condition with elevated serum CEA levels above the reference range (≥ 1 times the upper limit of normal).

### Statistical analysis

Quantitative variables were expressed as the means ± standard deviation (SD), and categorical variables were expressed as the frequencies and percentages. Comparisons between two groups were performed using Student’s *t-*test for quantitative variables and *χ*^*2*^ test for categorical variables. The incidence densities were calculated for MetS and Hyper-CEA in subcohort A and B, respectively. Cox proportional hazards models were performed to calculate adjusted hazard ratios (HRs) and 95% confidence intervals (CIs) in separate models for MetS in subcohort A and for Hyper-CEA in subcohort B. In subcohort A, data were adjusted for age, smoking, alcohol intake, the components of MetS, blood uric acid, alanine aminotransferase, aspartate aminotransferase, gamma-glutamyl transpeptidase, haemoglobin and white blood cell count. In subcohort B, data were adjusted for age, smoking, alanine aminotransferase, serum creatinine and white blood cell count. Stratified analysis across age groups (aged ≤45 years, > 45 years and ≤ 65 years and > 65 years) and smoking status (yes or no) was performed. Multivariable Cox proportional hazards models were also used to estimate the association between cigarette smoking and events in subcohorts A and B. The β, HR, HR 95% CI and *P* values were calculated to elucidate the effect of Hyper-CEA and MetS. Statistical analyses were performed using the SAS 9.4 software. All tests were two-sided. Statistical significance was defined as *P* < 0.05.

## Results

In subcohort A (from CEA to MetS), 1370 participants developed incident MetS from January 2010 to December 2015. The average follow-up time was 26.39 months (SD = 12.24). Participants with Hyper-CEA were older and had significantly higher SBP, DBP, FPG and WBC measures; lower BMI, triglyceride, ALT and blood uric acid values; and a greater percentage of smoking (Table 1). The incidence density of MetS was 84.56 per 1000 person-years among individuals with Hyper-CEA at baseline and 99.28 per 1000 person-years among those without (Table 2).

**Table 1:** Baseline characteristics of participants, means±SD or N (%)

Characteristics	Subcohort A			Subcohort B		
	Hyper-CEA (*N* = 551)	Norm-CEA (*N* = 5888)	*P*	MetS (*N* = 2451)	Non-MetS (*N* = 6082)	*P*
Age (year)	49.90 ± 14.25	42.13 ± 12.98	< 0.001	47.62 ± 12.9	42.37 ± 13.15	< 0.001
SBP (mmHg)	127.89 ± 16.96	125.52 ± 14.74	< 0.001	140.08 ± 15.85	125.74 ± 14.92	< 0.001
DBP (mmHg)	82.35 ± 11.23	81.11 ± 9.87	0.005	91.13 ± 10.75	81.24 ± 9.9	< 0.001
BMI (kg/m^2^)	24.11 ± 2.88	24.4 ± 2.85	0.025	27.68 ± 2.58	24.47 ± 2.87	< 0.001
FPG (mmol/l)	5.42 ± 1.40	5.18 ± 0.80	< 0.001	6.09 ± 1.54	5.19 ± 0.79	< 0.001
TG (mmol/l)	1.23 ± 0.79	1.30 ± 0.75	0.046	2.46 ± 1.58	1.31 ± 0.75	< 0.001
HDL-C (mmol/l)	1.42 ± 0.27	1.44 ± 0.25	0.218	1.28 ± 0.27	1.43 ± 0.25	< 0.001
ALT (U/L)	20.55 ± 10.27	23.14 ± 12.77	< 0.001	30.17 ± 16.08	23.22 ± 12.82	< 0.001
AST (U/L)	19.85 ± 6.92	20.21 ± 6.37	0.216	22.63 ± 8.03	20.19 ± 6.35	< 0.001
GGT (U/L)	29.15 ± 27.34	28.65 ± 22.00	0.622	46.92 ± 37.27	28.82 ± 21.75	< 0.001
BUN (mmol/l)	5.26 ± 1.19	5.24 ± 1.18	0.729	5.34 ± 1.17	5.24 ± 1.18	0.001
CREA (mmol/l)	77.39 ± 10.98	77.16 ± 10.18	0.612	76.38 ± 10.65	77.15 ± 10.21	0.002
BUA (umol/l)	347.45 ± 75.63	356.22 ± 70.55	0.006	386.16 ± 78.9	356.99 ± 71.16	< 0.001
HB (g/l)	153.84 ± 10.36	154.49 ± 9.80	0.145	156.32 ± 10.55	154.55 ± 9.86	< 0.001
WBC (10^9^/l)	6.73 ± 1.68	6.25 ± 1.41	< 0.001	6.77 ± 1.57	6.27 ± 1.41	< 0.001
Smoking			< 0.001			0.003
No (%)	262 (47.90)	3678 (62.75)		1442 (59.24)	3793 (62.67)	
Yes (%)	285 (52.10)	2183 (37.25)		992 (40.76)	2259 (37.33)	
Alcohol intake			0.112			0.004
No (%)	233 (42.60)	2293 (39.12)		870 (35.74)	2367 (39.11)	
Yes (%)	314 (57.40)	3568 (60.88)		1564 (64.26)	3685 (60.89)	
Overweight (%)	167 (30.31)	2224 (37.77)	< 0.001	2255 (92.00)	2361 (38.82)	< 0.001
Hyperglycaemia (%)	98 (17.79)	721 (12.25)	< 0.001	1479 (60.34)	752 (12.36)	< 0.001
Hypertension (%)	290 (52.63)	2499 (42.44)	< 0.001	2239 (91.35)	2605 (42.83)	< 0.001
Elevated triglycerides (%)	66 (11.98)	990 (16.81)	0.003	1791 (73.07)	1043 (17.15)	< 0.001
Reduced HDL-C (%)	16 (2.90)	103 (1.75)	0.054	379 (15.46)	118 (1.94)	< 0.001

*P* values were calculated by *t* test for quantitative variables and *χ*^*2*^ test for categorical variables. *CEA* Carcinoembryonic antigen; *Hyper-CEA* Elevated serum CEA level above the reference range; *Norm-CEA* Within the normal reference ranges; *MetS* Metabolic syndrome; *Non-MetS* Without metabolic syndrome; *SBP* Systolic blood pressure; *DBP* Diastolic blood pressure; *BMI* Body mass index; *FPG* Fasting plasma glucose; *TG* Triglyceride; *HDL-C* High-density lipoprotein cholesterol; *ALT* Alanine aminotransferase; *AST* Aspartate aminotransferase; *GGT* Gamma-glutamyl transpeptidase; *BUN* Blood urea nitrogen; *CREA* Serum creatinine; *BUA* Blood uric acid; *HB* Haemoglobin; *WBC* White blood cell count

**Table 2:** The follow-up information of subcohort A and subcohort B

Characteristics	Subcohort A (from CEA to MetS)		Subcohort B (from MetS to CEA)	
	High-CEA (*N* = 551)	Norm-CEA (*N* = 5888)	MetS (*N* = 2451)	Non-metS (*N* = 6082)
follow-up time (months)	22.94 ± 9.40	26.72 ± 12.42	28.24 ± 12.95	27.52 ± 12.56
incident frequency (%)	88 (15.97)	1282 (21.77)	185 (7.55)	411 (6.76)
incidence density (per 1000 person-years)	84.56	99.28	33.42	29.13

Subcohort A showed incident MetS in the High-CEA group and Norm-CEA group; subcohort B showed incident Hyper-CEA in the MetS group and Non-MetS group. *CEA* Carcinoembryonic antigen; *Hyper-CEA* elevated serum CEA level above the reference range; *Norm-CEA* Within the normal reference ranges; *MetS* Metabolic syndrome; *Non-MetS* Without metabolic syndrome

In subcohort B, 596 participants developed incident Hyper-CEA from January 2010 to December 2015, and the average follow-up time was 28.03 months (SD = 12.84). At baseline, participants with MetS were older; had significantly higher SBP, DBP, BMI, FPG, triglyceride, ALT, AST, gamma-glutamyl transpeptidase, blood urea nitrogen, blood uric acid, haemoglobin, WBC, smoking and alcohol intake; and lower HDL-C and serum creatinine levels (Table 1). The incidence densities of Hyper-CEA among individuals with or without MetS were 33.42 per 1000 person-years and 29.13 per 1000 person-years, respectively (Table 2).

In subcohort A, no significant effects of Hyper-CEA on incident MetS were observed (HR, 0.89; 95% CI, 0.71 to 1.12; *P* = 0.326) after adjusting for age, smoking, alcohol intake, the components of MetS, blood uric acid, ALT, AST, gamma-glutamyl transpeptidase, haemoglobin and white blood cell count (Table 3, Supplemental Table S3). In different age and smoking strata, Hyper-CEA did not have an effect on MetS, either (Table 4).

**Table 3:** Crude and adjusted hazard ratios (95% CI) of Hyper-CEA in MetS and MetS in Hyper-CEA

Characteristics	Model 1 ^a^			Model 2		
	*β*	Hazard ratio (95% CI)	*P*	*β*	Hazard ratio (95% CI)	*P*
Subcohort A						
Hyper-CEA	−0.059	0.94 (0.76, 1.18)	0.602	−0.116	0.89 (0.71, 1.12)^b1^	0.326
Norm-CEA		1			1	
Subcohort B						
MetS	0.161	1.17 (0.99, 1.40)	0.07	0.016	1.02 (0.84, 1.22)^b2^	0.864
Non-MetS		1			1	

^a^: Model 1, crude model without adjusting for any confounders; ^b1^: Model 2 in subcohort A, adjusted for age, smoking, alcohol intake, the components of MetS, blood uric acid, alanine aminotransferase, aspartate aminotransferase, gamma-glutamyl transpeptidase, haemoglobin and white blood cell count; ^b2^: Model 2 in subcohort B, adjusted for age, smoking, alanine aminotransferase, serum creatinine and white blood cell count. *CEA* Carcinoembryonic antigen; *Hyper-CEA* Elevated serum CEA level above the reference range; *Norm-CEA* Within the normal reference ranges; *MetS* Metabolic syndrome; *Non-MetS* Without metabolic syndrome

**Table 4:** The hazard ratio (95% CI) of Hyper-CEA for MetS in subcohort A by stratified analysis

Characteristics	Model 1^a^			Model 2^b^		
	*β*	Hazard ratio (95% CI)	*P*	*β*	Hazard ratio (95% CI)	*P*
Age ≤ 45 y, nonsmoking (*n* = 2498)						
Hyper-CEA	0.134	1.14 (0.69, 1.91)	0.607	−0.005	0.99 (0.58, 1.7)	0.985
Norm-CEA		1			1	
Age > 45 y and ≤ 65 y, nonsmoking(*n* = 1107)						
Hyper-CEA	−0.363	0.70 (0.41, 1.18)	0.18	−0.392	0.68 (0.39, 1.17)	0.161
Norm-CEA		1			1	
Age > 65 y, nonsmoking (*n* = 335)						
Hyper-CEA	−0.103	0.90 (0.46, 1.79)	0.768	−0.063	0.94 (0.45, 1.95)	0.867
Norm-CEA		1			1	
Age ≤ 45 y, smoking (*n* = 1527)						
Hyper-CEA	−0.169	0.84 (0.49, 1.46)	0.543	−0.223	0.8 (0.45, 1.42)	0.447
Norm-CEA		1			1	
Age > 45 y and ≤ 65 y, smoking (*n* = 855)						
Hyper-CEA	−0.185	0.83 (0.56, 1.23)	0.35	−0.019	0.98 (0.64, 1.51)	0.93
Norm-CEA		1			1	
Age > 65 y, smoking (*n* = 86)						
Hyper-CEA	−0.58	0.56 (0.12, 2.54)	0.452	0.128	1.14 (0.15, 8.66)	0.902
Norm-CEA		1			1	

^a^: Model 1 was the unadjusted hazard ratio; ^b^: Model 2 was adjusted for alcohol intake, the components of MetS, blood uric acid, alanine aminotransferase, aspartate aminotransferase, gamma-glutamyl transpeptidase, haemoglobin and white blood cell count. *CEA* Carcinoembryonic antigen; *Hyper-CEA* Elevated serum CEA level above the reference range; *Norm-CEA* Within the normal reference ranges; *MetS* Metabolic syndrome

In subcohort B, the association was similar when adjusting for different sets of confounders (Table 3). After adjusting for age, smoking, alanine aminotransferase, serum creatinine and WBC, the hazard ratio of MetS for incident Hyper-CEA was 1.02 (95% CI, 0.84 to 1.22, *P* = 0.864) compared without MetS at baseline (Table 3, Supplemental Table S4). Meanwhile, in the stratified analysis (Table 5), for participants aged > 65 years and nonsmoking, the adjusted hazard ratio of MetS for Hyper-CEA was 1.87 (95% CI, 1.09 to 3.20; *P* = 0.022).

**Table 5:** The hazard ratio (95% CI) of MetS for Hyper-CEA in subcohort B by stratified analysis

Characteristics	Model 1^a^			Model 2^b^		
	*β*	Hazard ratio (95% CI)	*P*	*β*	Hazard ratio (95% CI)	*P*
Age ≤ 45 y, nonsmoking (*n* = 3052)						
MetS	−0.081	0.92 (0.59, 1.44)	0.72	−0.165	0.85 (0.53, 1.35)	0.489
Non-MetS		1			1	
Age > 45 y and ≤ 65 y, nonsmoking (*n* = 1660)						
MetS	0.274	1.31 (0.92, 1.89)	0.137	0.338	1.40 (0.96, 2.05)	0.083
Non-MetS		1			1	
Age > 65 y, nonsmoking (*n* = 523)						
MetS	0.576	1.78 (1.08, 2.92)	0.023	0.626	1.87 (1.09, 3.20)	0.022
Non-MetS		1			1	
Age ≤ 45 y, smoking (*n* = 2000)						
MetS	−0.018	0.98 (0.66, 1.45)	0.929	−0.106	0.90 (0.59, 1.36)	0.616
Non-MetS		1			1	
Age > 45 y and ≤ 65 y, smoking (*n* = 1147)						
MetS	−0.332	0.72 (0.49, 1.06)	0.095	−0.316	0.73 (0.49, 1.09)	0.119
Non-MetS		1			1	
Age > 65 y, smoking (*n* = 104)						
MetS	−0.178	0.84 (0.3, 2.33)	0.733	−0.604	0.55 (0.14, 2.11)	0.38
Non-MetS		1			1	

^a^: Model 1 was the unadjusted hazard ratio; ^b^: Model 2 was adjusted for alanine aminotransferase, serum creatinine and white blood cell count. *CEA* Carcinoembryonic antigen; *Hyper-CEA* Elevated serum CEA level above the reference range; *MetS* Metabolic syndrome; *Non-MetS* Without metabolic syndrome

In the present study, smoking was an important risk factor for incident Hyper-CEA (adjusted hazard ratio, 1.55; 95% CI, 1.31 to 1.84; *P* < 0.001; Supplemental Table S4). The association between smoking and incident MetS disappeared after adjusting for other confounders (Supplemental Table S3). In subcohort A, after stratification of the adjusted models according to Hyper-CEA (yes or no), smoking showed no significant association with incident MetS (Table 6). In subcohort B, upon stratification according to MetS (yes or no), smoking without MetS was associated with incident Hyper-CEA (hazard ratio, 1.76; 95% CI, 1.43 to 2.15; *P* < 0.001) (Table 7).

**Table 6:** The hazard ratio (95% CI) of smoking among different CEA status in subcohort A

Characteristics	No. of patients with MetS	Event rate %	*β*	Hazard ratio for MetS^a^	*P*
Hyper-CEA (*n* = 547)					
Smoking	47	16.49	−0.064	0.94 (0.55, 1.59)	0.814
Nonsmoking	41	15.65		1	
Norm-CEA (*n* = 5861)					
Smoking	533	24.42	0.022	1.02 (0.9, 1.16)	0.742
Nonsmoking	742	20.17		1	

^a^: The hazard ratio was adjusted for age, alcohol intake, the components of MetS, blood uric acid, alanine aminotransferase, aspartate aminotransferase, gamma-glutamyl transpeptidase, haemoglobin and white blood cell count. *CEA* Carcinoembryonic antigen; *Hyper-CEA* Elevated serum CEA level above the reference range; *Norm-CEA* Within the normal reference ranges; *MetS* Metabolic syndrome

**Table 7:** The hazard ratio (95% CI) of smoking among different MetS statuses in subcohort B

Characteristics	No. of patients with Hyper-CEA	Event rate %	*β*	Hazard ratio for Hyper-CEA^a^	*P*
MetS (*n* = 2434)					
Smoking	75	7.56	0.165	1.18 (0.86, 1.62)	0.31
Nonsmoking	110	7.63		1	
Non-MetS (*n* = 6052)					
Smoking	205	9.07	0.563	1.76 (1.43, 2.15)	< 0.001
Nonsmoking	204	5.38		1	

^a^ The hazard ratio was adjusted for age, alanine aminotransferase, serum creatinine and white blood cell count. *CEA* Carcinoembryonic antigen; *Hyper-CEA* Elevated serum CEA level above the reference range; *MetS* Metabolic syndrome; *Non-MetS* Without metabolic syndrome

## Discussion

This is the first large-scale longitudinal cohort study to investigate the bidirectional association between CEA and MetS. In subcohort A, CEA showed no association with incident MetS. Meanwhile, in subcohort B, MetS in nonsmoking elderly men could increase the risk of incident Hyper-CEA.

Previous studies have shown that the index of CEA can increase the risk of many metabolic disorders using cross-sectional designs [9, 11, 12, 15, 19–21]. Moreover, a cross-sectional study also found a positive association between CEA and MetS based on data from 2014 to 2015 (Supplemental Table S2), and it was consistent with previous studies [15, 16]. However, according to subcohort A, CEA had no significant effect on incident MetS.

Cross-sectional studies do not permit distinction between cause and effect, and the advancement is not as good as a cohort study. Thus, the previous study could not assess the direction between CEA and MetS. In the present study, we found that CEA is not associated with incident MetS, but there may be a significant association from MetS to incident Hyper-CEA. On one hand, chronic low-grade inflammation plays a vital role in the pathogenesis and progression of insulin resistance, which underlies metabolic disorders [22, 23]. On the other hand, acute and chronic inflammation could also elevate CEA levels. The level of CEA is significantly associated with various inflammatory markers, such as leucocyte count, CRP, and IL-6 levels. This is because CEA can bind to the CEA receptors on macrophages or monocytes and stimulate the production of pro-inflammatory cytokines [21, 24].

Smoking status can increase incident Hyper-CEA [25], and smoking was a risk factor for incident Hyper-CEA in the present study. The general population may mask the effects of specific stratified populations. Thus, a stratified analysis was conducted. In subcohort B, MetS for nonsmoking elderly men could increase the risk of incident Hyper-CEA, while the association was not statistically significant for smoking elderly men. First, this may suggest that smoking is a major factor for Hyper-CEA. When smoking coexists with MetS, the effect of MetS on Hyper-CEA is weakened. Second, the sub-sample size of smoking elderly men might have been too small to detect the significant difference, and the sample size for smoking with MetS or Hyper-CEA needs to be increased. Third, many studies have found that MetS can increase the risk of colorectal cancer [2], and CEA has a role as a tumour marker for colorectal cancer [26]. There is a hypothesis that Hyper-CEA may be an intermediate status from MetS to cancer in nonsmoking elderly men. However, there is a lack of research on the role of Hyper CEA in the relationship between MetS and cancer. Further research is required to determine the link between MetS and incident Hyper-CEA.

### Study strength and limitations

This was the first large-scale longitudinal cohort study to investigate the association between CEA and MetS. Compared with a cross-sectional study design, a cohort study is a more useful tool to explore bidirectional associations. It also facilitated investigation of causality. Because potential causes are determined before the outcome in cohort studies, the debate over the time sequence of cause and effect can be avoided [27]. The second strength of this study was that two cohort studies (subcohort A and B) were simultaneously conducted to investigate the bidirectional association between CEA and MetS.

This study has several limitations. First, only health check-ups among northern Chinese males were included in the analyses. It may not be appropriate to extrapolate to other populations considering the role of genetic background in MetS. Second, the study did not include the change of variables during the follow-up, such as changes of life-style for participants. Third, the sample size of smoking with MetS or Hyper-CEA needs to be increased, and the follow-up period may need extending. This study will continue follow-ups in the future.

## Conclusion

The present study indicated that CEA has no association with incident MetS based on the cohort direction from CEA to MetS. For the effect of MetS on incident Hyper-CEA, MetS in nonsmoking elderly men had a significant effect on the risk of Hyper-CEA, while this association was not observed in other stratified participants. Furthermore, well-designed experimental or clinical studies are required to investigate the functional mechanisms underlying the association between CEA and MetS. The clinical implications of the association between CEA and MetS should be interpreted with caution.

## Supplementary Information

**Additional file 1 Supplemental Table S1.** Comparison of characteristics between participants with and without Hyper-CEA in a cross-sectional study (2014–2015). **Supplemental Table S2.** Crude and adjusted odds ratios (95% CI) of Hyper-CEA in MetS in a cross-sectional study (2014–2015). **Supplemental Table S3.** Crude and adjusted hazards ratios (95% CI) for incident MetS in subcohort A. **Supplemental Table S4.** Crude and adjusted hazards ratios (95% CI) for incident Hyper-CEA in subcohort B.

## Data Availability

The datasets used and/or analysed during the present study are available from the corresponding author on reasonable request.

## Abbreviations

CEA: Serum carcinoembryonic antigen
Hyper-CEA: Elevated serum CEA level above the reference range
Norm-CEA: Within the normal reference ranges of CEA
MetS: Metabolic syndrome
Non-MetS: Without metabolic syndrome
SD: Standard deviation
BMI: Body mass index
SBP: Systolic blood pressure
DBP: Diastolic blood pressure
FPG: Fasting plasma glucose
TC: Total cholesterol
TG: Triglyceride
LDL-C: Low-density lipoprotein-cholesterol
HDL-C: High-density lipoprotein cholesterol
HB: Haemoglobin
WBC: White blood cell count
BUA: Blood uric acid
GGT: Gamma-glutamyl transpeptidase
ALT: Alanine aminotransferase
AST: Serum aspartate aminotransferase
CREA: Serum creatinine
BUN: Blood urea nitrogen
HR: Hazards ratio
CI: Confidence interval
