# Supplementary material for: Bidirectional association between serum carcinoembryonic antigen and metabolic syndrome among the Chinese male population: two cohort studies
Source: Lipids Health Dis. 2020 Nov 4;19:233. doi: 10.1186/s12944-020-01411-7 (PMC7643476; doi:10.1186/s12944-020-01411-7)
Supplement: Supplementary file 1 — Additional file 1 Supplemental Table S1. Comparison of characteristics between participants with and without Hyper-CEA in a cross-sectional study (2014–2015). Supplemental Table S2. Crude and adjusted odds ratios (95% CI) of Hyper-CEA in MetS in a cross-sectional study (2014–2015). Supplemental Table S3. Crude and adjusted hazards ratios (95% CI) for incident MetS in subcohort A. Supplemental Table S4. Crude and adjusted hazards ratios (95% CI) for incident Hyper-CEA in subcohort B. [file 12944_2020_1411_MOESM1_ESM.docx]

**Supplemental material**

**Bidirectional association between serum carcinoembryonic antigen and metabolic syndrome among the Chinese male population: Two cohort Studies**

Supplemental Table S1. Comparison of characteristics between participants with and without Hyper-CEA in a cross-sectional study (2014-2015)

| **Characteristics** | **Hyper-CEA (N=1937)** | **Norm-CEA (N=15412)** | ***t/χ^2^*** | ***P* value** |
| --- | --- | --- | --- | --- |
| Age (year) | 51.83±14.35 | 43.94±13.48 | 581.401 | <0.001 |
| SBP (mmHg) | 134.26±19.66 | 130.66±16.83 | 75.453 | <0.001 |
| DBP (mmHg) | 85.36±12.24 | 84.19±11.59 | 17.299 | <0.001 |
| BMI (kg/m^2^) | 25.73±3.29 | 25.58±3.29 | 3.503 | 0.061 |
| FPG (mmol/l) | 6.04±2.18 | 5.48±1.25 | 286.74 | <0.001 |
| TG (mmol/l) | 1.56±0.97 | 1.54±0.96 | 0.363 | 0.547 |
| HDL-C (mmol/l) | 1.27±0.26 | 1.26±0.24 | 2.21 | 0.137 |
| ALT (U/L) | 22.86±12.23 | 24.89±14.26 | 35.736 | <0.001 |
| AST (U/L) | 20.7±7.15 | 20.71±6.95 | 0.001 | 0.973 |
| GGT (U/L) | 37.67±41.36 | 34.24±28.93 | 21.326 | <0.001 |
| BUN (mmol/l) | 5.12±1.23 | 5.00±1.16 | 17.206 | <0.001 |
| CREA (mmol/l) | 79.34±11.16 | 80.91±10.54 | 37.376 | <0.001 |
| BUA (umol/L) | 355.49±78.29 | 366.66±74.65 | 38.052 | <0.001 |
| HB (g/l) | 154.87±11.05 | 155.74±10.18 | 12.372 | <0.001 |
| WBC (10^9^ / l) | 7.02±1.72 | 6.69±1.56 | 76.053 | <0.001 |
| Age group |  |  |  | <0.001 |
| 20-45 | 674 (34.80) | 9194 (59.65) |  |  |
| 46-65 | 913 (47.13) | 5086 (33.00) |  |  |
| >65 | 350 (18.07) | 1132 (7.34) |  |  |
| Smoking |  |  | 132.147 | <0.001 |
| No (%) | 926 (48.18) | 9456 (61.79) |  |  |
| Yes (%) | 996 (51.82) | 5847 (38.21) |  |  |
| Alcohol intake |  |  | 0.007 | 0.936 |
| No (%) | 718 (37.40) | 5733 (37.49) |  |  |
| Yes (%) | 1202 (62.60) | 9559 (62.51) |  |  |
| Overweight (%) | 1121 (57.87) | 8640 (56.06) | 2.298 | 0.13 |
| Hyperglycemia (%) | 749 (38.67) | 4210 (27.32) | 108.622 | <0.001 |
| Hypertension (%) | 1268 (65.46) | 8932 (57.95) | 40.029 | <0.001 |
| Elevated triglycerides (%) | 620 (32.01) | 4808 (31.20) | 0.527 | 0.468 |
| Reduced HDL-C (%) | 266 (13.73) | 1905 (12.36) | 2.959 | 0.085 |
| MetS | 755 (38.98) | 4857 (31.51) | 43.799 | <0.001 |
| *P values were calculated by t test for quantitative variables and χ^2^ test for categorical variables. CEA: carcinoembryonic antigen; Hyper-CEA: elevated serum CEA level above the reference range; Norm-CEA: within the normal reference ranges of CEA; MetS: metabolic syndrome; SBP: systolic blood pressure; DBP: diastolic blood pressure; BMI: body mass index; FPG: fasting plasma glucose; TG: triglyceride; HDL-C: high-density lipoprotein cholesterol; ALT: alanine aminotransferase; AST: aspartate aminotransferase; GGT: gamma-glutamyl transpeptidase; BUN: blood urea nitrogen; CREA: serum creatinine; BUA: blood uric acid; HB: hemoglobin; WBC: white blood cell count. | | | | |

Supplemental Table S2. Crude and adjusted odds ratios (95% CI) of Hyper-CEA in MetS in a cross-sectional study (2014-2015)

| **Model** | **β** | **OR (95% CI)** | ***χ^2^*** | ***P* value** |
| --- | --- | --- | --- | --- |
| Model 1 ^a^ |  |  |  |  |
| Hyper-CEA | 0.328 | 1.39 (1.26 ,1.53) | 43.523 | < 0.001 |
| Norm-CEA | reference | | | |
| Model 2 ^b^ |  |  |  |  |
| Hyper-CEA | 0.171 | 1.19 (1.07 ,1.31) | 11.26 | 0.001 |
| Norm-CEA | reference | | | |
| Model 3 ^c^ |  |  |  |  |
| Hyper-CEA | 0.14 | 1.15 (1.04 ,1.27) | 7.327 | 0.007 |
| Norm-CEA | reference | | | |
| Model 4 ^d^ |  |  |  |  |
| Hyper-CEA | 0.118 | 1.13 (1.01 ,1.26) | 4.337 | 0.037 |
| Norm-CEA | reference | | | |

CEA: carcinoembryonic antigen; Hyper-CEA: elevated serum CEA level above the reference range; Norm-CEA: within the normal reference ranges of CEA; MetS: metabolic syndrome.

^a^: Model 1, crude model without adjusting for any confounders;

^b^: Model 2, adjusted for age;

^c^: Model 3, adjusted for age and smoking;

^d^: Model 4 in subcohort A, adjusted for age, smoking, alcohol intake, the components of MetS, hemoglobin, white blood cell count, blood uric acid, gamma-glutamyl transpeptidase, alanine aminotransferase, aspartate aminotransferase, serum creatinine, and blood urea nitrogen levels.

Supplemental Table S3. Crude and adjusted hazards ratios (95% CI) for incident MetS in subcohort A

| **Characteristics** | **Single Cox model** | | |  | **Multivariable Cox model** | | |
| --- | --- | --- | --- | --- | --- | --- | --- |
|  | **β** | **Hazard ratio (95% CI)** | ***P* value** |  | **β** | **Hazard ratio (95% CI)** | ***P* value** |
| Hyper-CEA | -0.059 | 0.94 (0.76, 1.18) | 0.602 |  | -0.116 | 0.89 (0.71, 1.12) | 0.326 |
| Age (year) |  |  |  |  |  |  |  |
| ≤ 45 y |  | reference | |  |  | reference | |
| > 45 y and ≤65 y | 0.377 | 1.46 (1.30, 1.63) | <0.001 |  | 0.316 | 1.37 (1.20, 1.56) | <0.001 |
| > 65 y | 0.173 | 1.19 (0.96, 1.47) | 0.114 |  | 0.358 | 1.43 (1.10, 1.85) | 0.007 |
| ALT (U/L) | 0.019 | 1.02 (1.02, 1.02) | <0.001 |  | 0.013 | 1.01 (1.01, 1.02) | <0.001 |
| AST (U/L) | 0.02 | 1.02 (1.01, 1.03) | <0.001 |  | -0.019 | 0.98 (0.97, 0.99) | 0.002 |
| GGT (U/L) | 0.009 | 1.01 (1.01, 1.01) | <0.001 |  | 0.006 | 1.01 (1.00, 1.01) | <0.001 |
| BUN (mmol/l) | 0.032 | 1.03 (0.99, 1.08) | 0.162 |  |  | -- | |
| CREA (mmol/l) | 0.003 | 1.00 (1.00, 1.01) | 0.286 |  |  | -- | |
| BUA (umol/L) | 0.004 | 1.00 (1.00, 1.00) | <0.001 |  | 0.002 | 1.00 (1.00, 1.00) | <0.001 |
| HB (g/l) | 0.01 | 1.01 (1.00, 1.02) | 0.001 |  | 0.002 | 1.00 (1.00, 1.01) | 0.508 |
| WBC (10^9^ / l) | 0.127 | 1.14 (1.10, 1.17) | <0.001 |  | 0.067 | 1.07 (1.03, 1.11) | 0.001 |
| Smoking | 0.193 | 1.21 (1.09, 1.35) | <0.001 |  | 0.021 | 1.02 (0.90, 1.16) | 0.739 |
| Alcohol intake | 0.209 | 1.23 (1.10, 1.38) | <0.001 |  | 0.098 | 1.10 (0.97, 1.25) | 0.129 |
| Overweight | 1.159 | 3.19 (2.86, 3.56) | <0.001 |  | 1.349 | 3.85 (3.38, 4.39) | <0.001 |
| Hyperglycemia | 0.509 | 1.66 (1.45, 1.91) | <0.001 |  | 0.956 | 2.60 (2.20, 3.08) | <0.001 |
| Hypertension | 0.362 | 1.44 (1.29, 1.60) | <0.001 |  | 0.666 | 1.95 (1.71, 2.22) | <0.001 |
| Elevated triglycerides | 0.678 | 1.97 (1.75, 2.22) | <0.001 |  | 0.91 | 2.48 (2.14, 2.89) | <0.001 |
| Reduced HDL-C | 0.947 | 2.58 (1.91, 3.48) | <0.001 |  | 1.574 | 4.83 (3.42, 6.81) | <0.001 |

CEA: carcinoembryonic antigen; Hyper-CEA: elevated serum CEA level above the reference range; MetS: metabolic syndrome; ALT: alanine aminotransferase; AST: aspartate aminotransferase; GGT: gamma-glutamyl transpeptidase; BUN: blood urea nitrogen; CREA: serum creatinine; BUA: blood uric acid; HB: haemoglobin; WBC: white blood cell count; HDL-C: high-density lipoprotein cholesterol.

Supplemental Table S4. Crude and adjusted hazards ratios (95% CI) for incident Hyper-CEA in subcohort B

| **Characteristics** | **Single Cox model** | | |  | **Multivariable Cox model** | | |
| --- | --- | --- | --- | --- | --- | --- | --- |
|  | **β** | **Hazard ratio (95% CI)** | ***P* value** |  | **β** | **Hazard ratio (95% CI)** | ***P* value** |
| MetS | 0.161 | 1.17 (0.99, 1.40) | 0.070 |  | 0.016 | 1.02 (0.84, 1.22) | 0.864 |
| Age (year) |  |  |  |  |  |  |  |
| ≤ 45 y |  | reference | |  |  | reference | |
| > 45 y and ≤65 y | 0.587 | 1.80 (1.51, 2.14) | <0.001 |  | 0.557 | 1.75 (1.46, 2.09) | <0.001 |
| > 65 y | 1.000 | 2.72 (2.13, 3.47) | <0.001 |  | 1.138 | 3.12 (2.39, 4.08) | <0.001 |
| ALT (U/L) | -0.006 | 0.99 (0.99, 1.00) | 0.055 |  | -0.003 | 1.00 (0.99, 1.00) | 0.376 |
| AST (U/L) | 0.006 | 1.01 (0.99, 1.02) | 0.311 |  |  | -- | |
| GGT (U/L) | 0.002 | 1.00 (1.00, 1.00) | 0.111 |  |  | -- | |
| BUN (mmol/l) | 0.040 | 1.04 (0.97, 1.11) | 0.251 |  |  | -- | |
| CREA (mmol/l) | -0.010 | 0.99 (0.98, 1.00) | 0.015 |  | -0.010 | 0.99 (0.98, 1.00) | 0.013 |
| BUA (umol/L) | -0.001 | 1.00 (1.00, 1.00) | 0.226 |  |  | -- | |
| HB (g/l) | -0.004 | 1.00 (0.99, 1.00) | 0.318 |  |  | -- | |
| WBC (10^9^ / l) | 0.097 | 1.10 (1.05, 1.16) | <0.001 |  | 0.084 | 1.09 (1.03, 1.15) | 0.002 |
| Smoking | 0.383 | 1.47 (1.25, 1.72) | <0.001 |  | 0.440 | 1.55 (1.31, 1.84) | <0.001 |
| Alcohol intake | -0.004 | 1.00 (0.84, 1.18) | 0.963 |  |  | -- | |

CEA: carcinoembryonic antigen; Hyper-CEA: elevated serum CEA level above the reference range; MetS: metabolic syndrome; ALT: alanine aminotransferase; AST: aspartate aminotransferase; GGT: gamma-glutamyl transpeptidase; BUN: blood urea nitrogen; CREA: serum creatinine; BUA: blood uric acid; HB: haemoglobin; WBC: white blood cell count.
